# TXNDC12 promotes EMT and metastasis of hepatocellular carcinoma cells via activation of β-catenin

**DOI:** 10.1038/s41418-019-0421-7

**Published:** 2019-09-30

**Authors:** Kefei Yuan, Kunlin Xie, Tian Lan, Lin Xu, Xiangzheng Chen, Xuefeng Li, Mingheng Liao, Jiaxin Li, Jiwei Huang, Yong Zeng, Hong Wu

**Affiliations:** 1 Department of Liver Surgery & Liver Transplantation, State Key Laboratory of Biotherapy and Cancer Center, West China Hospital, Sichuan University and Collaborative Innovation Center of Biotherapy, Chengdu, China; 2 Laboratory of Liver Surgery, West China Hospital, Sichuan University, Chengdu, China; 3 Shenzhen Luohu People’s Hospital, The Third Affiliated Hospital of Shenzhen University, Shenzhen, Guangdong China

**Keywords:** Metastasis, Oncogenes

## Abstract

Metastasis is one of the main contributors to the poor prognosis of hepatocellular carcinoma (HCC). However, the underlying mechanism of HCC metastasis remains largely unknown. Here, we showed that TXNDC12, a thioredoxin-like protein, was upregulated in highly metastatic HCC cell lines as well as in portal vein tumor thrombus and lung metastasis tissues of HCC patients. We found that the enforced expression of TXNDC12 promoted metastasis both in vitro and in vivo. Subsequent mechanistic investigations revealed that TXNDC12 promoted metastasis through upregulation of the ZEB1-mediated epithelial–mesenchymal transition (EMT) process. We subsequently showed that TXNDC12 overexpression stimulated the nuclear translocation and activation of β-catenin, a positive transcriptional regulator of ZEB1. Accordingly, we found that TXNDC12 interacted with β-catenin and that the thioredoxin-like domain of TXNDC12 was essential for the interaction between TXNDC12 and β-catenin as well as for TXNDC12-mediated β-catenin activation. Moreover, high levels of TXNDC12 in clinical HCC tissues correlated with elevated nuclear β-catenin levels and predicted worse overall and disease-free survival. In summary, our study demonstrated that TXNDC12 could activate β-catenin via protein–protein interaction and promote ZEB1-mediated EMT and HCC metastasis.

## Introduction

Liver cancer, of which 70–90% are hepatocellular carcinoma (HCC), is the second leading cause of cancer death worldwide [1]. Although many advances have been achieved in the diagnosis and treatment of HCC, the overall prognosis for HCC patients remains poor [2]. Tumor metastasis contributes greatly to the poor prognosis of HCC patients. The current therapeutic strategies for HCC have been demonstrated to promote HCC metastasis instead of repressing it [3]. Therefore, investigations of the molecular mechanisms underlying HCC metastasis are urgently needed to develop potential therapeutic strategies to target HCC metastasis.

Epithelial–mesenchymal transition (EMT) is a highly conserved cellular program during which epithelial cells lose their polarized organization and acquire migratory and invasive capabilities [4]. For the invasion of most tumor cells originating from solid tumors into adjacent cell layers, these cells must undergo EMT to lose cell–cell adhesion and acquire motility. Thus, EMT has been recognized as a prometastatic cellular event that promotes tumor cell invasion and malignant tumor progression [5]. Although recent studies have raised concerns about the actual contribution of EMT to metastasis in lung cancer [6] and pancreatic cancer [7], the experimental results acquired in HCC still support the idea that EMT plays a pivotal role in HCC metastasis [8–11]. Loss of E-cadherin, a typical marker of epithelial cells, is one of the key features of the EMT process and has been implicated in tumor metastasis [12]. Recent studies have shown that a series of transcription factors are direct repressors of E-cadherin, including β-catenin, a key component of the canonical Wnt signaling pathway [13, 14]. Interestingly, β-catenin is a dual-function protein that is implicated in transcriptional regulation as well as in cell–cell adhesion. In normal epithelial cells, most β-catenin forms an adhesion complex with E-cadherin and is located in cell-cell adherent junctions at the membrane. In tumor cells, a portion of the β-catenin detaches from the complex and translocates into the nucleus to perform its transcriptional regulatory function. However, the molecular mechanisms by which β-catenin detaches from the membrane E-cadherin/β-catenin complex and translocates into the nucleus remain largely unknown.

Thioredoxin domain-containing protein 12 (TXNDC12), which is also known as ERp16, ERp18, ERp19, or hTLP19, is a member of the protein disulfide isomerase (PDI) family that plays important roles in cancer development and progression [15–17]. TXNDC12 has been demonstrated to contribute to tumorigenicity in human gastric cancer by promoting cell growth, migration and invasion [18]. Meanwhile, another study revealed a contradictory finding that low mRNA levels of TXNDC12 predict poor prognosis in lung adenocarcinoma patients [19]. However, the role of TXNDC12 in HCC remains unknown.

In this study, we found that TXNDC12 was upregulated in HCC and correlated with HCC metastasis. Enforced expression of TXNDC12 stimulated the metastatic potential of HCC cells via induction of the EMT process. In addition, we revealed that TXNDC12 interacted with and activated β-catenin, thereby contributing to ZEB1 upregulation and EMT. Moreover, we showed a positive correlation between TXNDC12 and the nuclear expression of β-catenin, as well as the prognostic potential of this combination. Taken together, our data indicate that the overexpression of TXNDC12 promotes HCC metastasis through β-catenin-mediated induction of EMT and that TXNDC12 has the potential to be a prognostic factor and therapeutic target of HCC.

## Results

### TXNDC12 is upregulated in HCC and correlates with HCC metastasis

**Fig. 1:**
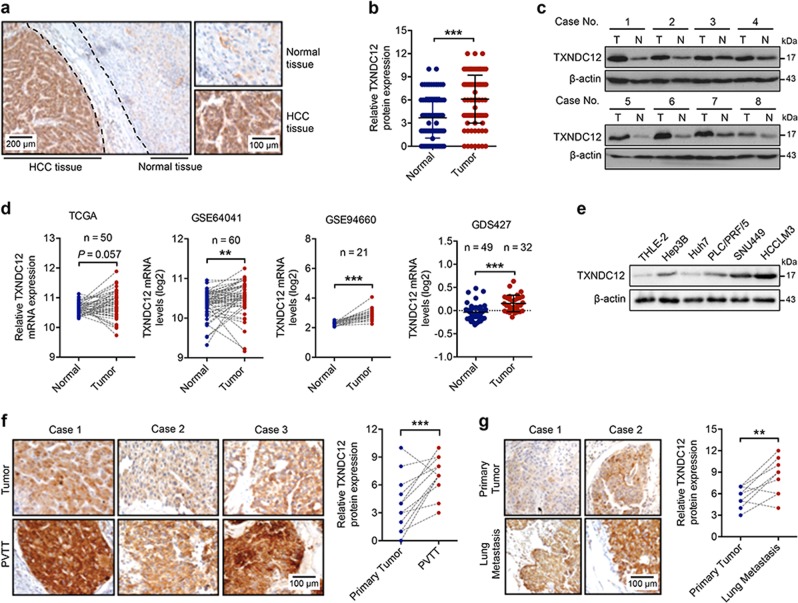
TXNDC12 is upregulated in human hepatocellular carcinoma. **a** Representative immunohistochemical staining of TXNDC12 in human hepatocellular carcinoma (HCC) tissues; scale bars: 40 μm (left), 20 μm (right). **b** Quantification of TXNDC12 expression in human HCC tissues (*n* = 106). The data are the means ± SDs. **c** Representative images of TXNDC12 expression obtained by immunoblot analysis in HCC tissues. **d** Relative mRNA expression of TXNDC12 in the TCGA dataset and three GEO datasets (GSE64041, GSE94660, and GDS427). The data are the means ± SDs. **e** Relative protein expression of TXNDC12 in normal hepatocytes and four HCC cell lines, as obtained by immunoblot analysis. **f** Representative images and quantification of TXNDC12 expression in portal vein tumor thrombus and matched primary HCC tissues obtained by immunohistochemical analysis (*n* = 14); scale bar: 20 μm. **g** Representative images and quantification of TXNDC12 expression in lung metastases and matched primary HCC tissues obtained by immunohistochemical analysis (*n* = 10); scale bar: 20 μm. **P < 0.01; ***P < 0.001

To clarify the underlying role of TXNDC12 in HCC, the expression of TXNDC12 was evaluated in 106 paired human HCC samples (Supplementary Table 1). Immunohistochemical staining revealed increased expression of TXNDC12 in HCC samples compared with that in adjacent normal liver tissues (Fig. 1a, b). Similar results were also observed in the immunoblot analyses (Fig. 1c). In addition, upregulated expression of TXNDC12 in HCC was confirmed by gene expression analysis based on a dataset from The Cancer Genome Atlas (TCGA) and three Gene Expression Omnibus (GEO) datasets (Fig. 1d). To explore the role of TXNDC12 in HCC progression, we examined the expression levels of TXNDC12 in one human normal hepatocyte cell line, THLE-2, and four human HCC cell lines, Hep3B, Huh7, PLC/PRF/5, and HCCLM3. The results showed that the expression levels of TXNDC12 were lowest in normal hepatocytes (THLE-2) and were significantly higher in the highly metastatic cell lines (SNU449 and HCCLM3) than in weakly metastatic cell lines (Hep3B, Huh7, and PLC/PRF/5) (Fig. 1e). Portal vein tumor thrombus (PVTT) is closely related to HCC metastasis and is correlated with poor prognosis of HCC patients [20]. To further explore the role of TXNDC12 in HCC metastasis, we first compared the expression levels of TXNDC12 between PVTT tissues and the corresponding patient-matched primary tumor tissues. As shown in Fig. 1f, the protein levels of TXNDC12 were significantly higher in PVTT tissues than in their counterpart tumor tissues. Moreover, we examined the expression levels of TXNDC12 in HCC lung metastases and the corresponding patient-matched primary tumors. Similarly, the protein levels of TXNDC12 were higher in HCC lung metastases than in their counterpart tumor tissues (Fig. 1g). Taken together, these results showed that TXNDC12 is frequently upregulated in HCC, particularly in metastatic lesions, suggesting that TXNDC12 may promote HCC metastasis.

### Upregulation of TXNDC12 promotes HCC cell motility and metastasis both in vitro and in vivo

**Fig. 2:**
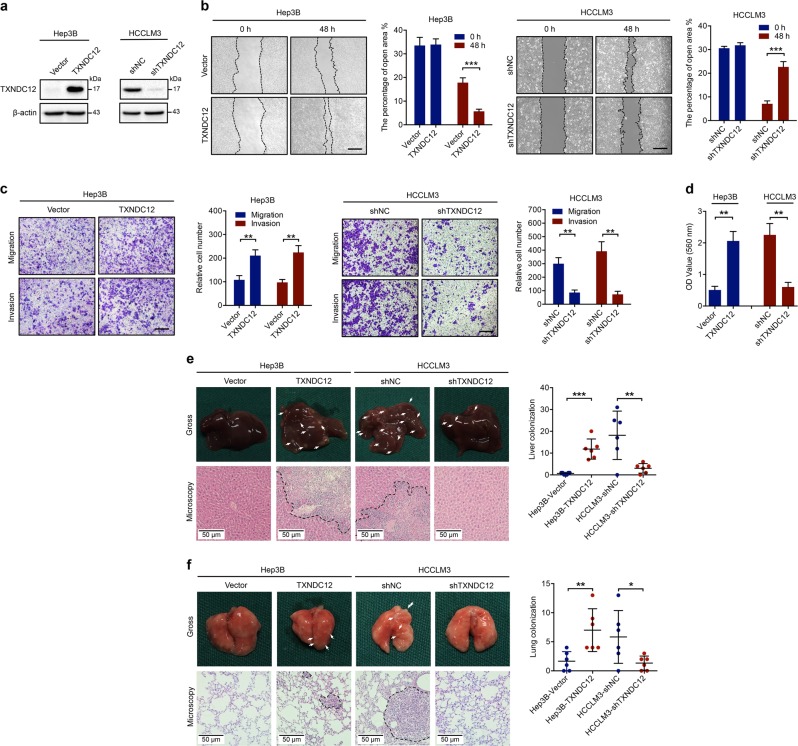
TXNDC12 enhances the metastatic potential of HCC cells. **a** Overexpression of TXNDC12 in Hep3B cells and knockdown of TXNDC12 in HCCLM3 cells, as confirmed by immunoblot analysis. **b** Representative data from wound healing migration assays performed with the indicated HCC cells. Scale bar = 200 μm. The data are the means ± SDs and are representative of three independent experiments. **c** Representative data from Transwell migration and Matrigel invasion assays performed with the indicated HCC cells. Scale bar = 200 μm. The data are the means ± SDs and are representative of three independent experiments. **d** Representative data from the cell adhesion assay performed with the indicated HCC cells. The data are the means ± SDs and are representative of three independent experiments. **e** Representative images (gross, HE, and IHC of TXNDC12) and the quantification of liver metastasis nodules, as well as the tumoral expression of TXNDC12, from the orthotopic HCC models established by injection of the indicated cells (*n* = 6 per group). **f** Representative images (gross, HE, and IHC of TXNDC12) and quantification of lung metastasis nodules, as well as the tumoral expression of TXNDC12, from the lung metastasis models established by injection of the indicated cells (*n* = 6 per group). *P < 0.05; **P < 0.01; ***P < 0.001

To elucidate the functions of TXNDC12 in HCC metastasis, we overexpressed TXNDC12 in Hep3B cells, which exhibited relatively low endogenous TXNDC12 levels. In addition, we knocked down TXNDC12 in HCCLM3 cells, which exhibited relatively high endogenous TXNDC12 levels (Fig. 2a). The wound healing migration, transwell migration, and matrigel invasion assays revealed that the overexpression of TXNDC12 enhanced the migration and invasion ability of HCC cells, whereas inhibition of TXNDC12 reduced the motility of HCC cells (Fig. 2b, c). In addition, we performed a fibronectin cell adhesion assay to examine the adhesion of tumor cells to the extracellular matrix, which permitted subsequent invasion and metastasis of the tumor cells. The results showed that the enforced expression of TXNDC12 increased the adhesion ability of HCC cells, while knockdown of TXNDC12 inhibited HCC cell adhesion (Fig. 2d). Similarly, knockdown of TXNDC12 by specific siRNAs repressed cell migration, invasion, and adhesion in SNU449 cells (Supplementary Fig. 1a–d).

To determine whether TXNDC12 had the same effect on HCC metastasis in vivo, an orthotopic HCC model was established. As shown in Fig. 2e, overexpression of TXNDC12 significantly promoted liver colonization of Hep3B cells, whereas depletion of TXNDC12 significantly inhibited liver colonization of HCCLM3 cells. We next generated a lung metastasis mouse model by tail vein injection of the aforementioned cells. A higher number of metastatic nodules were observed in mice injected with the Hep3B-TXNDC12 cells than in those injected with Hep3B-Vector cells, while fewer metastatic nodules were observed in mice injected with HCCLM3-shTXNDC12 cells than in those injected with HCCLM3-shNC cells (Fig. 2f). Collectively, these results indicate that TXNDC12 is capable of promoting the invasion and metastasis of HCC cells both in vitro and in vivo.

### Upregulation of TXNDC12 promoted EMT in HCC cells

**Fig. 3:**
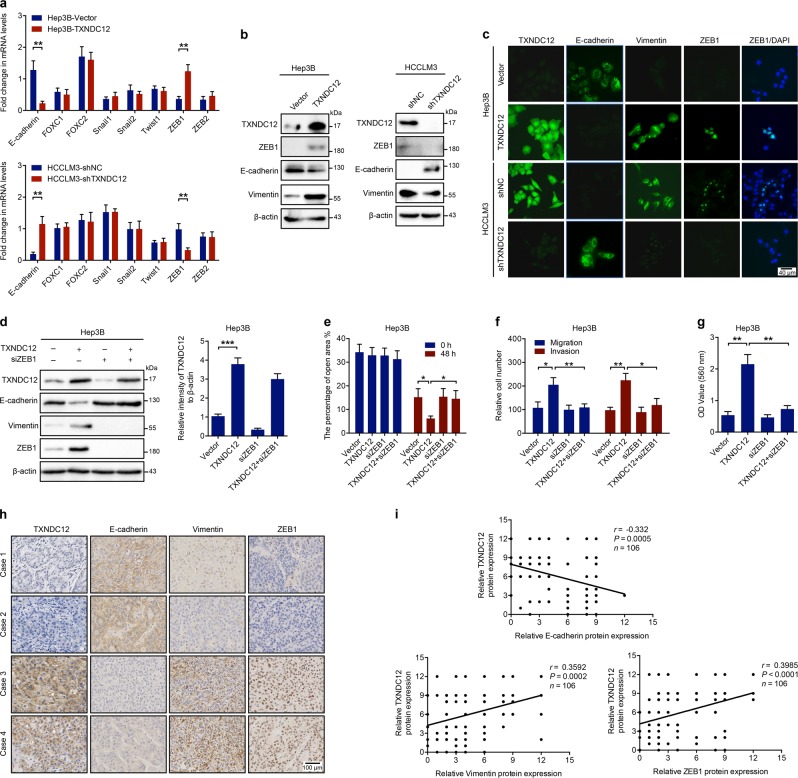
TXNDC12 promotes EMT in HCC cells via upregulation of ZEB1. **a** Fold change in the mRNA levels of E-cadherin, FOXC1, FOXC2, Snail1, Snail2, Twist1, ZEB1, and ZEB2 in the indicated cells. The data are the means ± SDs and are representative of three independent experiments. **b** Relative expression levels of TXNDC12, ZEB1, E-cadherin, and Vimentin in the indicated cells. **c** TXNDC12, E-cadherin, Vimentin, and ZEB1 expression in the indicated cells, as detected by an immunofluorescence assay. The merged images show overlays of ZEB1 (green) and nuclear staining by DAPI (blue); scale bar: 40 mm. **d** Relative expression levels of TXNDC12, E-cadherin, Vimentin, and ZEB1 in the indicated cells treated with or without siZEB1. **e** Wound healing migration assays performed with the indicated HCC cells treated with or without siZEB1. The data are the means ± SDs and are representative of three independent experiments. **f** Transwell migration and Matrigel invasion assays performed with the indicated HCC cells treated with or without siZEB1. The data are the means ± SDs and are representative of three independent experiments. **g** Representative data from the cell adhesion assay performed with the indicated HCC cells treated with or without siZEB1. The data are the means ± SDs and are representative of three independent experiments. **h** Representative immunohistochemical staining of TXNDC12, E-cadherin, Vimentin, and ZEB1 in human HCC tissues; scale bar: 40 μm. **i** Correlation analysis between TXNDC12 and E-cadherin, Vimentin or ZEB1 expression in liver tumor tissues from patients with HCC (*n* = 106). *P < 0.05; **P < 0.01

To explore the underlying molecular mechanisms, we first examined whether EMT, which is recognized as a key process of HCC metastasis [4], was involved in TXNDC12-mediated upregulation of HCC metastasis. The mRNA expression levels of E-cadherin and a series of EMT inducers, including FOXC1, FOXC2, Snail1, Snail2, Twist1, ZEB1, and ZEB2, were measured in both TXNDC12-overexpressing and TXNDC12 knockdown cells. Overexpression of TXNDC12 decreased E-cadherin mRNA levels and increased ZEB1 (an inducer of EMT and a key transcriptional repressor of E-cadherin [21]) mRNA levels, while knockdown of TXNDC12 increased E-cadherin and decreased ZEB1 mRNA levels (Fig. 3a). No significant change was observed in the expression levels of the other EMT inducers investigated upon overexpression or knockdown of TXNDC12. We then performed immunoblot analyses to confirm that TXNDC12 could inhibit the protein expression of E-cadherin and upregulate that of ZEB1, as well as that of Vimentin, a mesenchymal cell marker (Fig. 3b). Consistent with the previous results, immunofluorescence staining showed a clear increase in ZEB1 and Vimentin expression as well as a decrease in E-cadherin expression in TXNDC12-overexpressing HCC cells relative to the corresponding expression levels in the control group (Fig. 3c). In addition, we observed that in the aforementioned mouse models, enforced expression of TXNDC12 increased the expression levels of ZEB1, while knockdown of TXNDC12 decreased the expression levels of ZEB1 (Fig. 2e, f and Supplementary Fig. 2a, b). Therefore, these data suggested that TXNDC12 might be an inducer of EMT in HCC cells.

To verify whether TXNDC12 induced EMT in HCC cells through upregulation of ZEB1, we knocked down ZEB1 expression in TXNDC12-overexpressing cells and observed that ZEB1 knockdown eliminated the TXNDC12-mediated upregulation of Vimentin and downregulation of E-cadherin (Fig. 3d). Consistent with this finding, the EMT-mediated enhancement of cell motility was abolished by ZEB1 knockdown (Fig. 3e–g). These results demonstrated that ZEB1 was crucial for the TXNDC12-induced EMT process in HCC cells.

We then assessed the clinical relationship between TXNDC12 and EMT in HCC tissues. Immunohistochemical staining showed that HCC patients with low TXNDC12 expression showed higher E-cadherin and lower Vimentin expression levels, as well as lower ZEB1 expression levels, than HCC patients with high TXNDC12 expression (Fig. 3h, Supplementary Fig. 3a–c). Moreover, correlation analyses showed that the expression of TXNDC12 correlated with the expression of E-cadherin (*r* = -0.332, *P* = 0.005), Vimentin (*r* = 0.3592, *P* = 0.002), and ZEB1 (*r* = 0.3985, *P* < 0.001) (Fig. 3i). Taken together, these results demonstrated that TXNDC12 plays a vital role in EMT in HCC.

### TXNDC12 promotes the ZEB1-mediated EMT process through β-catenin activation

**Fig. 4:**
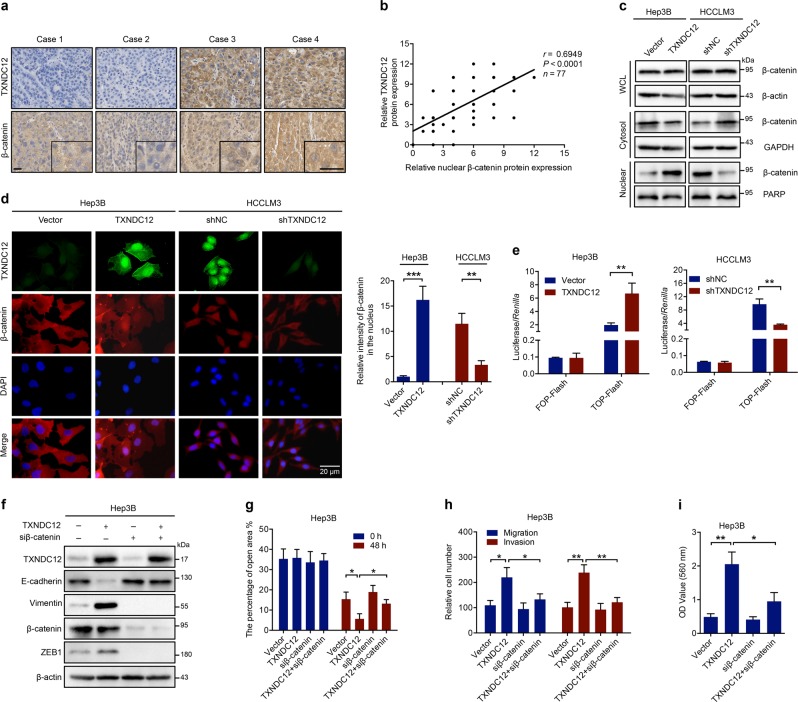
TXNDC12 upregulates ZEB1 through activation of β-catenin. **a** Representative immunohistochemical staining of TXNDC12 and β-catenin in human HCC tissues; scale bars: 20 μm (left), 20 μm (right). **b** Correlation analysis between TXNDC12 and nuclear β-catenin expression in liver tumor tissues from patients with HCC (*n* = 77). **c** β-Catenin expression in whole-cell lysates and the cytoplasmic, and nuclear fractions, as detected by immunoblot analysis. **d** β-Catenin expression in the indicated cells, as detected by an immunofluorescence assay. The merged images show overlays of β-catenin (red) and nuclear staining by DAPI (blue); scale bar: 20 mm. **e** TOP-Flash/FOP-Flash assay showing β-catenin activity in the indicated HCC cells. **f** Relative expression levels of TXNDC12, E-cadherin, Vimentin, β-catenin, and ZEB1 in the indicated cells treated with or without siβ-catenin. **g** Wound healing migration assays performed with the indicated HCC cells treated with or without siβ-catenin. The data are the means ± SDs and are representative of three independent experiments. **h** Transwell migration and Matrigel invasion assays performed with the indicated HCC cells treated with or without siβ-catenin. The data are the means ± SDs and are representative of three independent experiments. **i** Representative data from the cell adhesion assay performed with the indicated HCC cells treated with or without siβ-catenin. The data are the means ± SDs and are representative of three independent experiments. *P < 0.05; **P < 0.01

β-Catenin plays a critical role in the induction of EMT during HCC metastasis [22], and transcription of ZEB1 has been demonstrated to be upregulated by β-catenin [23]. To determine whether β-catenin is involved in TXNDC12-mediated ZEB1 upregulation, we assessed the clinical relationship between TXNDC12 and β-catenin in HCC tissues. Immunohistochemical staining showed that HCC patients with low TXNDC12 expression displayed lower nuclear β-catenin expression levels than HCC patients with high TXNDC12 expression (Fig. 4a, Supplementary Fig. 3d). Consistent with this finding, correlation analyses showed that the protein expression of TXNDC12 was closely associated with that of nuclear β-catenin (*r* = 0.6949, *P* < 0.001) (Fig. 4b). In addition, we found that in the aforementioned mouse models, enforced expression of TXNDC12 increased the nuclear β-catenin expression levels, while knockdown of TXNDC12 decreased the nuclear β-catenin expression levels (Fig. 2e, f and Supplementary Fig. 2a, b). These results suggested that TXNDC12 might promote the nuclear translocation of β-catenin.

To verify this finding, the cytosolic and nuclear fractions of cell lysates were separated. Neither overexpression nor knockdown of TXNDC12 significantly affected the total β-catenin level (Fig. 4c). However, TXNDC12 overexpression decreased the level of cytosolic β-catenin and increased the level of nuclear β-catenin, while knockdown of TXNDC12 showed the opposite effect on the subcellular distribution of β-catenin (Fig. 4c). In addition, we then performed immunofluorescence analysis to further investigate the subcellular location of β-catenin. Similar to the above findings, β-catenin was localized primarily in the cytoplasm of Hep3B-Vector and HCCLM3-shTXNDC12 cells, which have relatively low levels of TXNDC12 expression; however, the levels of nuclear β-catenin were significantly enhanced in Hep3B-TXNDC12 and HCCLM3-shNC cells, which show relatively high levels of TXNDC12 expression (Fig. 4d). Next, the transcriptional activity of β-catenin was evaluated by transfection of TOP-Flash and FOP-Flash luciferase reporters into the aforementioned HCC cells. As shown in Fig. 4e, the transcriptional activity of β-catenin was stimulated by TXNDC12 overexpression, whereas knockdown of TXNDC12 repressed the transcriptional activity of β-catenin.

To determine whether the activation of β-catenin is essential for the TXNDC12-mediated promotion of EMT, we knocked down the expression of β-catenin in TXNDC12-overexpressing cells and observed that knockdown of β-catenin eliminated the TXNDC12-mediated upregulation of ZEB1 and Vimentin, as well as the TXNDC12-mediated downregulation of E-cadherin (Fig. 4f). Consistent with this finding, the EMT-mediated upregulation of cell invasion and migration was abolished by β-catenin knockdown (Fig. 4g–i), suggesting that β-catenin plays a vital role in the TXNDC12-induced EMT process.

To further confirm the role of TXNDC12 in the EMT process, TGFβ1 was used to induce EMT in HCC cells. As shown in Supplementary Fig. 4a, treatment with TGFβ1 promoted the nuclear translocation of β-catenin, elevated the transcriptional activity of β-catenin, decreased the expression of E-cadherin, and increased the levels of Vimentin and ZEB1, indicating that EMT was induced by TGFβ1 treatment (Supplementary Fig. 4a–c). However, TGFβ1-induced EMT was significantly attenuated by inhibition of TXNDC12 (Supplementary Fig. 4a–c). Similarly, we found that inhibition of TXNDC12 abrogated the increase in cell motility induced by TGFβ1 (Supplementary Fig. 4d–f). Taken together, these results demonstrated that TXNDC12 overexpression leads to β-catenin activation, thereby leading to the upregulation of ZEB1 and promotion of the EMT process.

### TXNDC12 binds β-catenin and negatively regulates the formation of the E-cadherin/β-catenin complex

**Fig. 5:**
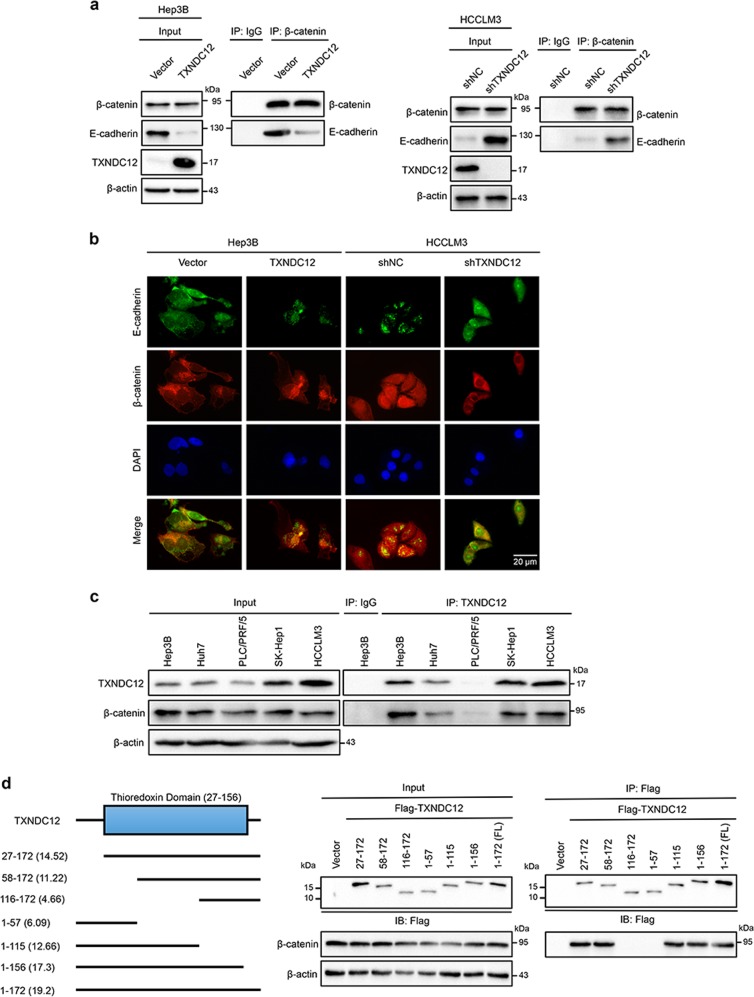
TXNDC12 interacts with β-catenin via the thioredoxin-like domain. **a** Immunoprecipitation of β-catenin with E-cadherin in the indicated HCC cells, as detected by immunoblot analysis. **b** E-cadherin and β-catenin expression in the indicated cells, as detected by an immunofluorescence assay. The merged images show overlays of E-cadherin (green) and β-catenin (red). The nuclei were stained by DAPI (blue). **c** Total cell lysates from five HCC cell lines were immunoprecipitated with an anti-TXNDC12 antibody or control IgG. The β-catenin and TXNDC12 levels are indicated. **d** 293T cells transfected with Flag-tagged full-length TXNDC12 or TXNDC12 fragments were immunoprecipitated with an anti-Flag antibody. The β-catenin and Flag-TXNDC12 (full-length or fragments) levels are indicated

The formation of the E-cadherin/β-catenin complex has been demonstrated to negatively regulate the activation of β-catenin [24]. To explore the mechanism by which TXNDC12 induces β-catenin activation, we examined the formation of the E-cadherin/β-catenin complex by co-IP and immunofluorescence analysis. As shown in Fig. 5a, b, the amount of E-cadherin/β-catenin complex in HCC cells with high TXNDC12 expression was lower than that in HCC cells with low TXNDC12 expression. We also found that endogenous β-catenin and TXNDC12 coimmunoprecipitated in multiple HCC cell lines (Fig. 5c), suggesting that TXNDC12 may activate β-catenin through protein–protein interaction. Subsequently, we mapped the binding domains of TXNDC12 by transfection of TXNDC12 truncation mutants into 293T cells. Only the fragments containing the active site of the thioredoxin domain (58–115) interacted with β-catenin (Fig. 5d), indicating that the thioredoxin domain of TXNDC12 is essential for the interaction between TXNDC12 and β-catenin.

### Mutation of the cysteines in the active site of TXNDC12 blocks β-catenin activation

**Fig. 6:**
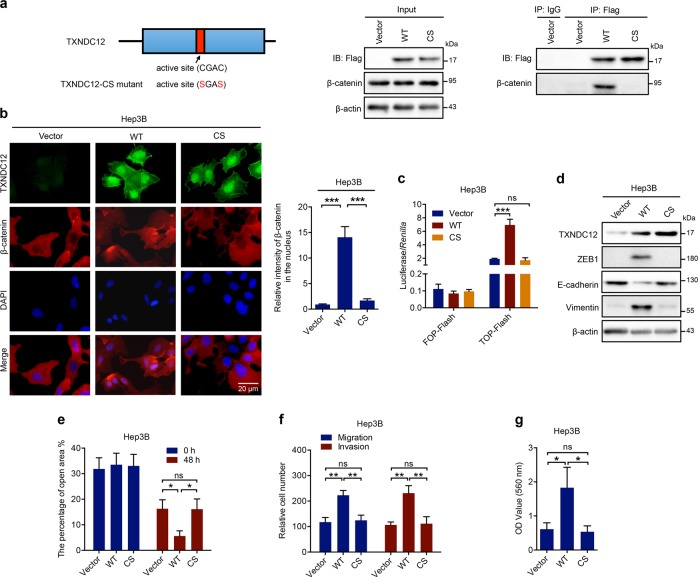
Mutation of the cysteines in the active site of TXNDC12 blocks its interaction with β-catenin and inhibits β-catenin activation. **a** 293T cells transfected with Flag-tagged vector, wild-type TXNDC12 (WT), or cysteine-mutated TXNDC12 (CS) were immunoprecipitated with an anti-Flag antibody. The β-catenin and Flag-TXNDC12 (full-length or fragment) levels are indicated. **b** β-Catenin expression in the indicated cells, as detected by immunofluorescence assay. The merged images show overlays of β-catenin (red) and nuclear staining by DAPI (blue); scale bar: 20 mm. **c** TOP-Flash/FOP-Flash assay showing β-catenin activity in the indicated HCC cells. **d** Relative expression levels of TXNDC12, ZEB1, E-cadherin, and Vimentin in the indicated cells. **e** Wound healing migration assays performed with the indicated HCC cells. The data are the means ± SDs and are representative of three independent experiments. **f** Transwell migration and Matrigel invasion assays performed with the indicated HCC cells. The data are the means ± SDs and are representative of three independent experiments. **g** Representative data from the cell adhesion assay performed with the indicated HCC cells. The data are the means ± SDs and are representative of three independent experiments. *P < 0.05; **P < 0.01; ***P < 0.001

To further investigate whether the PDI enzymatic activity of TXNDC12 is required for β-catenin activation, we constructed a TXNDC12-CS mutant in which both cysteines in the active site were mutated. As shown in Fig. 6a, the interaction between TXNDC12 and β-catenin was eliminated by the mutation. Consistent with this result, we found that the TXNDC12-CS mutant was unable to induce the nuclear translocation of β-catenin (Fig. 6b). Similarly, the transcriptional activity of β-catenin in HCC cells transfected with the TXNDC12-CS mutant was not significantly different from that in control cells (Fig. 6c). We also found that the TXNDC12-CS mutant did not induce either the EMT process (Fig. 6d) or cell motility (Fig. 6e–g). Taken together, these results demonstrated that the active site of TXNDC12 is crucial for TXNDC12-mediated β-catenin activation.

### Upregulation of TXNDC12 predicts poor survival in HCC patients

**Fig. 7:**
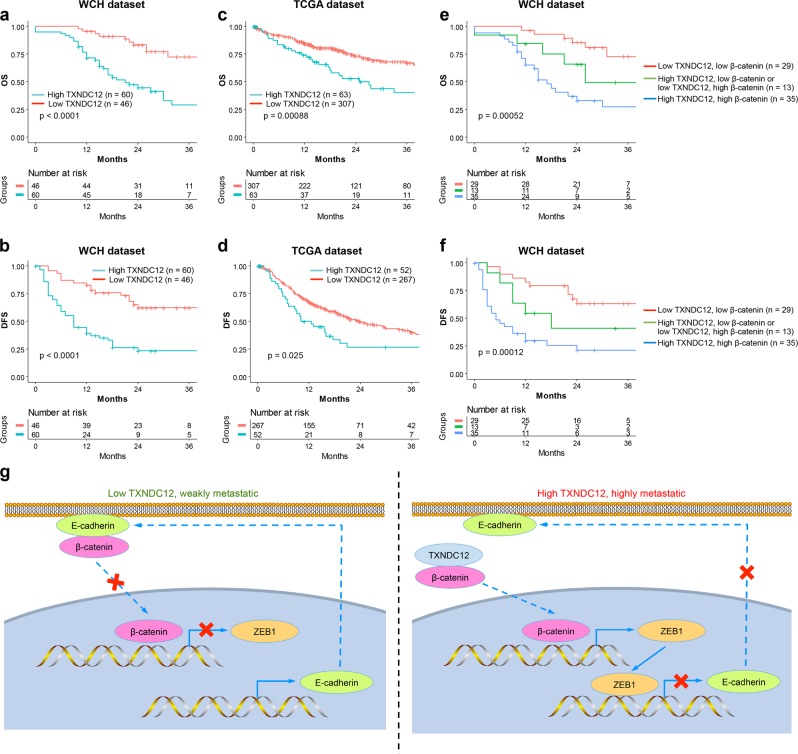
High levels of TXNDC12 predict poor prognosis in HCC patients. **a** Cumulative overall survival curves of patients from the West China Hospital (WCH) dataset with high or low TXNDC12 expression levels. **b** Cumulative disease-free survival curves of patients from the WCH dataset with high or low TXNDC12 expression levels. **c** Cumulative overall survival curves of patients from the TCGA dataset with high or low TXNDC12 expression levels. **d** Cumulative disease-free survival curves of patients from the TCGA dataset with high or low TXNDC12 expression levels. **e** Cumulative overall survival curves stratified by the combination of TXNDC12 and nuclear β-catenin expression levels. HCC patients were classified into three groups according to TXNDC12 and nuclear β-catenin expression: group 1 (*n* = 29): low TXNDC12 and β-catenin expression; group 2 (*n* = 13): high TXNDC12 and low β-catenin expression or low TXNDC12 and high β-catenin expression; group 3 (*n* = 35): high TXNDC12 and β-catenin expression. **f** Cumulative disease-free survival curves stratified by the combination of TXNDC12 and nuclear β-catenin expression levels. **g** A schematic model showing the function of TXNDC12 in regulating β-catenin activation and EMT. Elevated TXNDC12 levels promote the nuclear translocation of β-catenin through protein–protein interaction. β-Catenin activates the transcription of ZEB1, which then suppresses the expression of E-cadherin and induces EMT, ultimately contributing to HCC metastasis

To further investigate the clinical significance of TXNDC12 expression in HCC, all 106 HCC patients were divided into two groups based on the overall expression level of TXNDC12: the high TXNDC12 expression group (*n* = 60) and the low TXNDC12 expression group (*n* = 46). As shown in Supplementary Table 2, the upregulation of TXNDC12 was significantly correlated with several aggressive clinicopathological characteristics, such as poor differentiation (*P* < 0.001), multiple tumors (*P* = 0.006), a lack of encapsulation (*P* < 0.001), high serum α-fetoprotein (AFP) levels ( ≥ 20 ng/mL; *P* < 0.001), microvascular invasion (*P* = 0.003), and macrovascular invasion (*P* = 0.019). For both the West China Hospital (WCH) and TCGA datasets, the unadjusted survival curves showed that the high TXNDC12 expression group had worse OS (hazard ratio (HR), 3.80; 95% confidence interval (CI), 1.91–7.54) and DFS (HR, 3.54; 95% CI, 1.98–6.36) than the low TXNDC12 expression group (*P* < 0.05). The 3-year OS rate of the low TXNDC12 expression group was higher than that of the high TXNDC12 expression group. Similarly, the 3-year DFS rate of the low TXNDC12 expression group was higher than that of the high TXNDC12 expression group (Fig. 7a–d; Supplementary Table 3). Moreover, the expression levels of nuclear β-catenin were significantly higher in PVTT tissues than in the corresponding patient-matched primary tumor tissues, while the expression levels of E-cadherin were lower in PVTT tissues than in their counterpart tumor tissues (Supplementary Fig. 5a, b). Similarly, the protein levels of nuclear β-catenin were higher in HCC lung metastases than in their counterpart tumor tissues, while the protein levels of E-cadherin were lower in HCC lung metastases than in their counterpart tumor tissues (Supplementary Fig. 5c, d). These results further confirmed that TXNDC12 could stimulate the nuclear translocation of β-catenin and subsequently downregulate the expression of E-cadherin. Interestingly, we found that patients with low expression of both TXNDC12 and nuclear β-catenin had the best prognosis; in contrast, patients with high expression of both TXNDC12 and nuclear β-catenin had the worst prognosis (Fig. 7e, f). Taken together, these results indicated that the combination of TXNDC12 and nuclear β-catenin could serve as a biomarker in HCC for evaluating the metastatic potential and predicting the prognosis of HCC patients.

## Discussion

Metastasis is one of the major obstacles in HCC therapy. Few HCC patients with distant metastasis have the opportunity for surgery, and most systematic treatments have been demonstrated to be unsuitable for treating HCC patients [2]. Sorafenib has long been the only treatment strategy for advanced HCC patients. However, this treatment has been demonstrated to promote metastasis rather than blocking it [3]. Recently, three novel drugs, regorafenib, lenvatinib, and nivolumab (a PD-1 inhibitor), have been approved by the FDA for HCC treatment. Among these drugs, the first two are less likely to inhibit HCC metastasis because they are both multikinase inhibitors similar to sorafenib. The effect of nivolumab on HCC metastasis remains to be determined. Therefore, there is currently no effective treatment for HCC metastasis. In this study, we identified TXNDC12 as a novel regulator upstream of β-catenin. Since β-catenin plays a vital role in HCC metastasis but has been defined as an undruggable target [2], identification of the upstream factors of β-catenin may provide potential therapeutic targets. However, the potential of TXNDC12 as a drug target requires further evaluation.

Recent studies have demonstrated that PDI family members play important roles in cancer development and progression. ERp5, which is highly expressed in the lymph node microenvironment, promotes immune escape through blockade of NKG2D ligand recognition in Hodgkin lymphoma [25]. TXNDC5 directly interacts with the androgen receptor protein to increase its stability and enhance its transcriptional activity, thus stimulating the growth of castration-resistant prostate cancer [26]. Moreover, a small molecule targeting PDI has been shown to trigger the chemosensitization of cancer cells [27], which further supports the importance of PDIs in cancer. A member of the PDI family, TXNDC12 has been implicated in the tumorigenesis and metastasis of gastric cancer [18]. However, the role of TXNDC12 in HCC remains unknown. In this study, we found that TXNDC12 induced the migration and invasion of HCC cells in vitro. In addition, we observed that TXNDC12 promoted intrahepatic metastasis and lung colonization of HCC cells in mouse models. More importantly, we elucidated the molecular mechanisms by which TXNDC12 regulates HCC metastasis. Our findings will facilitate the understanding of the functions of PDIs and the evaluation of PDIs as biomarkers or therapeutic targets for cancer.

Accumulating evidence has demonstrated that EMT is one of the key processes in tumor metastasis [28–30]. However, the most recent advances in EMT research have questioned the requirement for EMT in tumor metastasis [6, 7]. In one study, pancreatic ductal adenocarcinoma (PDAC) mouse models were established with deletion of Snail or Twist, two key transcription factors responsible for EMT. However, the results showed that EMT suppression in the primary tumor does not alter PDAC metastasis [7]. In another study, an EMT lineage-tracing system was established, and the results showed that within a predominantly epithelial primary tumor, only a small proportion of tumor cells undergo EMT. Notably, lung metastases mainly consist of non-EMT tumor cells. Moreover, inhibiting EMT by overexpressing the microRNA miR-200 did not affect the development of lung metastasis [6]. Therefore, these two studies proved that EMT is not required for the metastasis of all tumors. In our study, we demonstrated that TXNDC12 upregulated the expression of ZEB1 and induced the EMT process. Since we proved that TXNDC12 promoted the migration and invasion of HCC cells, we hypothesized that TXNDC12 might stimulate HCC metastasis through the promotion of EMT. To exclude the possibility that EMT is dispensable for TXNDC12-induced HCC metastasis, we utilized a specific siRNA targeting ZEB1, a key transcription factor involved in EMT, and concluded that EMT was required for TXNDC12-induced HCC metastasis.

The Wnt/β-catenin pathway is one of the key regulatory pathways in cancer development and progression. It has been widely reported that the Wnt/β-catenin pathway plays crucial roles in hepatocarcinogenesis and metastasis [31–33]. β-Catenin is a key factor in the Wnt/β-catenin pathway, and the expression of β-catenin was found to be upregulated in HCC tissues compared with that in adjacent normal liver tissues. In addition, β-catenin mutations are frequently identified in HCC, suggesting that the function of β-catenin may be vital for the development and progression of HCC. Our study showed that TXNDC12 promoted EMT through upregulation of ZEB1 expression. Since a previous study proved that β-catenin is an upstream regulator of ZEB1 [23], we hypothesized that TXNDC2 might induce ZEB1 expression through regulation of the Wnt/β-catenin pathway. Then, we demonstrated that TXNDC12 activated the Wnt/β-catenin pathway. Although TXNDC12 did not enhance the total expression levels of β-catenin, it promoted the nuclear translocation of β-catenin, thereby stimulating the transcription of β-catenin downstream genes, including ZEB1. TXNDC12 is a member of the PDI family that is located in the cytoplasm and functions via protein–protein interactions. Interestingly, β-catenin was also present in the cytoplasm, suggesting that TXNDC12 might promote the nuclear translocation of β-catenin through direct protein–protein interaction. To verify this hypothesis, we confirmed the interaction between TXNDC12 and β-catenin by immunoprecipitation. Collectively, the results of our study linked TXNDC12 with β-catenin, thus clarifying the underlying mechanism of TXNDC12-mediated HCC metastasis. However, the detailed mechanisms by which the interaction between TXNDC12 and β-catenin promotes the nuclear translocation of β-catenin require further investigation.

In summary, our study demonstrated a novel role of TXNDC12 in the regulation of EMT via interaction with β-catenin and transcriptional activation of ZEB1 (Fig. 7g). Overexpression of TXNDC12 in HCC is a strong indicator of high tumor aggressiveness and correlates with poor clinical outcomes. Investigation of the biological functions and the underlying molecular mechanisms of TXNDC12 in HCC have shed light on our understanding of the EMT process and HCC metastasis. In conclusion, our study identified an important PDI, TXNDC12, that plays a vital role in HCC metastasis and could be a potential prognostic biomarker and therapeutic target for HCC.

## Materials and methods

### Cell lines

Hep3B, Huh7, PLC/PRF/5, and HCCLM3 cells were obtained from the Cell Bank Type Culture Collection of the Chinese Academy of Sciences, Shanghai, China. THLE-2 and SNU449 cells were obtained from ATCC (Manassas, VA, USA). All cells were cultivated in Dulbecco’s modified Eagle’s medium (HyClone, Logan, UT, USA) containing 10% FBS (HyClone), penicillin (10^7^ U/L), and streptomycin (10 mg/L) at 37 °C and 5% CO_2_.

### Clinical samples

All HCC tissues, corresponding adjacent normal tissues, and HCC PVTT and lung metastasis tissues were obtained from deidentified patients who had undergone surgical resection at West China Hospital (WCH; Chengdu, China). A total of 106 tumor tissues and paired adjacent normal tissues were collected from patients who underwent resection for HCC at WCH between 2010 and 2014. The detailed protocols for tissue collection and clinical data processing (including age, sex, hepatitis B or C virus infection status, preoperative serum AFP level, tumor differentiation grade, the number of nodules, the presence of vascular invasion, and the Ishak fibrosis score of the adjacent liver tissue) were performed as described previously [34]. The study was approved by the local ethics committee of WCH. The detailed clinicopathologic features are listed in Supplementary Table 1.

### TOP/FOP Flash assay

Cells were plated in 24-well plates at 2 × 10^4^ cells per well 24 h before transfection. Cells were transfected with Lef/Tcf luciferase reporter (TOP-Flash, Upstate) or FOP-Flash reporter (Upstate) plus the pRL-CMV plasmid (Renilla luciferase, Promega). After 48 h, luciferase activity was measured using a dual luciferase reporter assay system (Promega) following the manufacturer’s protocol.

### In vivo models

For the mouse lung metastasis model, 2 × 10^6^ cells were injected into nude mice (BALB/c, 6 weeks of age, *n* = 6 per group) (HFK Bio, Beijing, China) through the tail vein. After 4 weeks, mice were sacrificed, and the lungs were excised, imaged, and embedded in paraffin. The orthotopic mouse model of HCC was established as described previously [35]. Briefly, mice were anesthetized, and 2 × 10^6^ cells were injected into the subcapsular region of the middle lobe. After 6 weeks, mice were sacrificed, and the livers were excised, imaged, and embedded in paraffin. Tumor metastases were confirmed by hematoxylin and eosin staining. All animal experiments were approved by the Institutional Animal Care and Use Committee of Sichuan University.

### TCGA and GEO data analysis

Level 3 RNA-sequencing data and clinical information for 370 HCC patients were downloaded from the cBioPortal for Cancer Genomics (www.cbioportal.org). X-tile plots (version 3.6.1, Yale University School of Medicine) were used to select the optimum cutoff for the expression of TXNDC12 based on the association of gene mRNA levels with the patients’ OS. Microarray gene expression data of HCC patient cohorts were downloaded from the GEO database (accession numbers GSE64041, GSE94660, and GDS427). The R package “GEOquery” was used to extract the expression values of the genes of interest.

### Statistical analysis

Statistical analyses were conducted and graphics were generated using Prism version 6.00 (GraphPad Software) and R software (R 3.3.2). Cox proportional hazards models were used to evaluate the association of TXNDC12 and β-catenin with overall and disease-specific mortality. One-way ANOVA and the Pearson *χ*^2^ test were used for univariate analyses. The log-rank test was used to compare patient survival between subgroups. The Kaplan–Meier method was used to calculate survival rates. All statistical tests were two-sided, and *P* < 0.05 was considered statistically significant.

## Supplementary information

Supplementary Information
