# Supplementary material for: TXNDC12 promotes EMT and metastasis of hepatocellular carcinoma cells via activation of β-catenin
Source: Cell Death Differ. 2019 Sep 30;27(4):1355–68. doi: 10.1038/s41418-019-0421-7 (PMC7206186; doi:10.1038/s41418-019-0421-7)
Supplement: Supplementary file 1 — Supplementary Information [file 41418_2019_421_MOESM1_ESM.docx]

**Supplementary Information**

**Supplementary Materials and Methods**

**Constructs, transfection and reagents.** The full-length and truncated TXNDC12 plasmid (Flag-TXNDC12) was constructed by inserting a PCR-amplified full-length or truncated fragment into the pCMV-Flag vector (Sigma). The TXNDC12 mutant construct (both Cys66 and Cys69 were replaced by serine, CS) was generated with the QuikChange XL site-directed mutagenesis kit (Stratagene). Lentiviral vector containing TXNDC12 gene was constructed by GenePharma (Shanghai, China). Lentiviral particles containing shTXNDC12 were from Santa Cruz (sc-60597). Specific siRNAs targeting ZEB1 (J-006564-10-0005) and β-catenin (J-003482-10-0005) were from Dharmacon. Specific siRNAs targeting TXNDC12 were from

Viewsolid Biotech (Beijing, China). The sequences were as follows：

TXNDC12 siRNA-1 sense: GCAAAGCUCUAAAGCCCAATT;

TXNDC12 siRNA-1 antisense: UUGGGCUUUAGAGCUUUGCTT;

TXNDC12 siRNA-2 sense: GGAUGAAGAGGAACCCAAATT;

TXNDC12 siRNA-2 antisense: UUUGGGUUCCUCUUCAUCCTT;

TXNDC12 siRNA-3 sense: GCAAGGUGCAUCCUGAAAUTT;

TXNDC12 siRNA-3 antisense: AUUUCAGGAUGCACCUUGCTT;

For plasmid and siRNA transfection, cells were transient transfected with Lipofectamine 3000 and Lipofectamine RNAiMAX transfection reagent, respectively (Invitrogen). Recombinant human TGFβ1 protein was purchased from Abcam (ab50036).

**Wound-healing migration assay.** For wound-healing migration assay, confluent monolayers of cells were wounded with a p20 pipette tip. The images were taken after PBS wash (time 0 h). The cells were cultured in serum-free medium. After two days, the images were taken again (time 48 h). Three separate fields were photographed for each plate (one of three fields was shown in the figure).

**Transwell migration and Matrigel invasion assays.** Transwell membrane (8 μm pore size, 6.5 mm diameter; Corning Costar) was used for both assays. For transwell migration assay, 2.5×10^4^ cells were plated in the top chambers. The top chambers were filled with serum-free medium and the bottom chambers were filled with migration-inducing medium (with 10% FBS). The filters were fixed with 4% paraformaldehyde after 24 h. The cells on the upper side of the membrane were scraped with a cotton swab and the cells on the bottom side of the membrane were stained with crystal violet. The membranes were washed with PBS and photographed after dry out. For Matrigel invasion assay, the top chambers were coated with Matrigel before 2.5×10^4^ cells were plated. Images were taken after 72 h.

**Cell adhesion assay.** Cell adhesion assay was performed using the CytoSelect 48-Well Cell Adhesion Assay kit (fibronectin coated) following the manufacturer’s protocol (Cell Biolabs, San Diego, CA). Briefly, cell suspension containing 1.5×10^5^ cells was plated into each well and incubated for 60 min in the cell culture incubator. Then the media was carefully removed and the well was washed. After that, the cells were stained and lysed. The extraction of the cells were measured in a plate reader at OD = 560 nm.

**Real-time PCR.** Total RNA was extracted from cells using Trizol (Invitrogen). cDNA was prepared using Superscript II reverse transcriptase (Invitrogen). Real-time PCR was performed using SYBR Green PCR kit (Takara) according to the manufacturer’s instructions. The primer sequences were as follows：

E-cadherin Forward: AGCCCCGCCTTATGATTCTCTG；

E-cadherin Reverse: TGCCCCATTCGTTCAAGTAGTCAT；

Snail1 Forward: GACCCCAATCGGAAGCCTAACTAC；

Snail1 Reverse: AGCCTTTCCCACTGTCCTCATC；

Snail2 Forward: CCTCCATCTGACACCTCC；

Snail2 Reverse: CCCAGGCTCACATATTCC；

Twist1 Forward: CTCAAGAGGTCGTGCCAATC；

Twist1 Reverse: CCCAGTATTTTTATTTCTAAAGGTGTT；

ZEB1 Forward: AAGTGGCGGTAGATGGTA；

ZEB1 Reverse: TTGTAGCGACTGGATTTT；

ZEB2 Forward: TTCTGCGACATAAATACG；

ZEB2 Reverse: GAGTGAAGCCTTGAGTGC；

FOXC1 Forward: CAGCATCCGCCACAACCTCT；

FOXC1 Reverse: GCAGCCTGTCCTTCTCCTCCT；

FOXC2 Forward: GCCTAAGGACCTGGTGAAGC；

FOXC2 Reverse: TTGACGAAGCACTCGTTGAG；

GAPDH Forward: GGAGCGAGATCCCTCCAAAAT；

GAPDH Reverse: GGCTGTTGTCATACTTCTCATGG.

**Immunoblot analysis.** The cells were lysed in RIPA buffer (Beyotime Biotechnology, China) and quantified using the BCA protein assay kit (Beyotime Biotechnology, China). Primary antibodies were from Santa Cruz (β-actin, 1:1000, mouse monoclonal, sc-69879; E-cadherin, 1:1000, mouse monoclonal, sc-8426; Vimentin, 1:1000, mouse monoclonal, sc-6260), Zen Bioscience (GAPDH, 1:1000, mouse monoclonal, 200306), Thermo Fisher (TXNDC12, 1:1000, Rabbit Polyclonal, PA5-24798), Cell Signaling Technology (PARP, 1:1000, Rabbit Polyclonal, 9542; ZEB1, 1:1000, Rabbit Polyclonal, 3396; β-catenin, 1:1000, Rabbit Polyclonal, 8480; Flag tag, 1:1000, Rabbit Polyclonal, 14793). Secondary antibodies were from Santa Cruz (Goat anti-mouse IgG-HRP, 1:5000, sc-2005; Goat anti-rabbit IgG-HRP, 1:5000, sc-2004). For preparation of cytoplasmic and nuclear proteins, NE-PER nuclear and cytoplasmic extraction reagents (Pierce) were used according to the manufacturer’s instructions. GAPDH was used as a loading control for the cytoplasmic fraction, and PARP was used as a loading control for the nuclear fraction. Immunoprecipitation was performed using Pierce classic IP kit (Thermo Fisher) according to the manufacturer’s instructions. Briefly, a total of 200 μg cell lysate were suspended in 500 μl of RIPA buffer and incubated with antibodies at 4°C for 12 hours, followed by addition of 100 μl protein A/G plus Agarose for 4 hours. Supernatants of the immunoprecipitation reaction were collected to determine β-actin as a loading control.

**Immunohistochemistry.** Tissues were formalin-fixed and paraffin-embedded. The sections were consecutively cut (4 μm thickness). Immunohistochemistry (IHC) analysis was performed as described previously(35). The staining score was calculated according to the immunostaining intensity and the percentage of positive HCC cells.

**Immunofluorescence analysis.** Cells were grown on coverslips in a 24-well plate (MatTek, Ashland, MA). For immunostaining, the cells were fixed in 4% paraformaldehyde, permeabilized with 0.2% Triton X-100 in PBS, and blocked with blocking buffer (5% BSA in PBS) for 1 h. Cells were incubated with primary antibodies diluted 1:100 in blocking buffer for 1 h and washed three times with PBST (PBS with 0.1% Tween-20). After incubation with appropriate fluorophore-conjugated secondary antibodies (Molecular Probes) and DAPI (Thermo Fisher), the coverslips were mounted on slides. The images were captured by AX10 imager A2 microscope (Carl Zeiss MicroImaging), and processed using the software provided by the manufacturer.

**
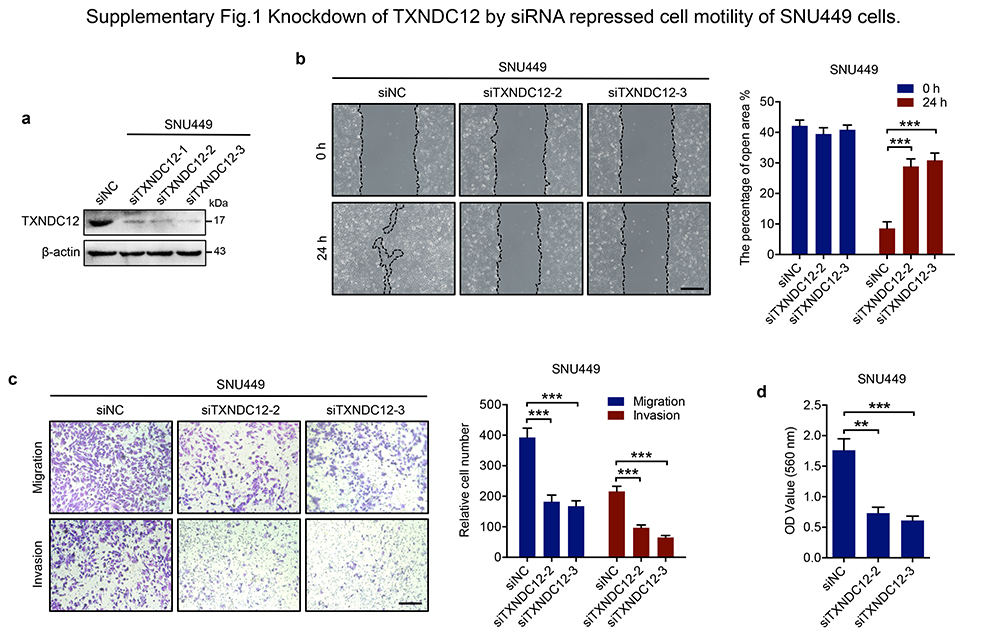
**

**Supplementary Fig. 1** Knockdown of TXNDC12 by siRNA repressed cell motility of SNU449 cells. **a** Knockdown of TXNDC12 in SNU449 cells by siRNAs, as confirmed by immunoblot analysis. **b** Representative data from wound healing migration assays performed with the indicated HCC cells. Scale bar = 200 μm. The data are the means ± SDs and are representative of three independent experiments. **c** Representative data from Transwell migration and Matrigel invasion assays performed with the indicated HCC cells. Scale bar = 200 μm. The data are the means ± SDs and are representative of three independent experiments. **d** Representative data from the cell adhesion assay performed with the indicated HCC cells. The data are the means ± SDs and are representative of three independent experiments. ***P* < 0.01; ****P* < 0.001.

**
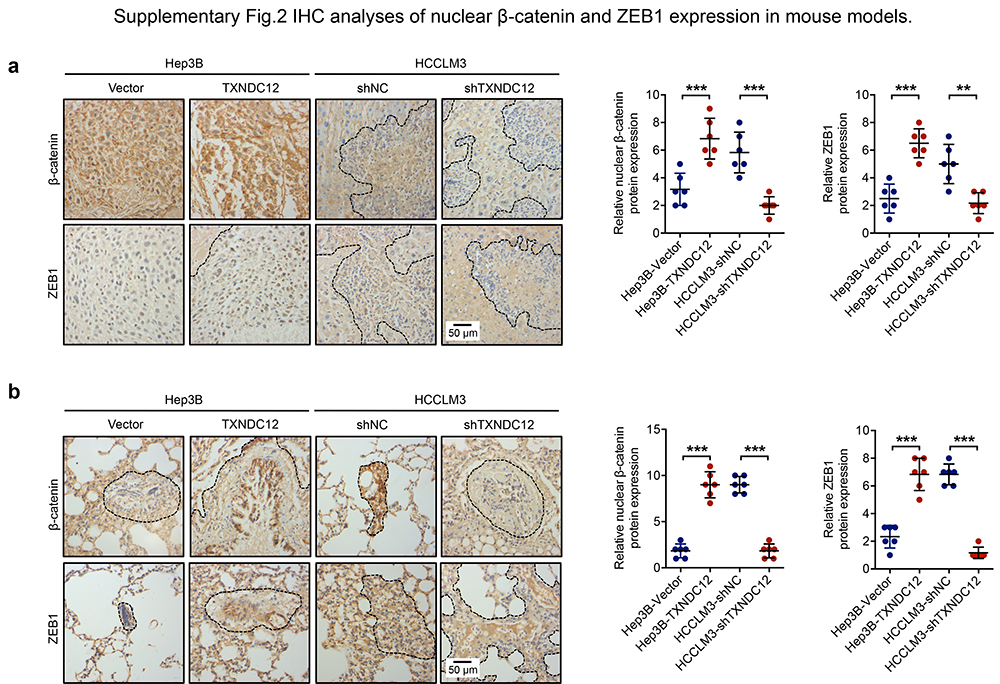
Supplementary Fig. 2** IHC analyses of nuclear β-catenin and ZEB1 expression in mouse models. **a** Representative images (IHC of β-catenin and ZEB1) and quantification of nuclear β-catenin and ZEB1 expression from the orthotopic HCC models generated by indicated cells (n = 6 for each group). **b** Representative images (IHC of β-catenin and ZEB1) and quantification of nuclear β-catenin and ZEB1 expression from the lung metastasis models generated by indicated cells (n = 6 for each group). ***P* < 0.01; ****P* < 0.001.

**
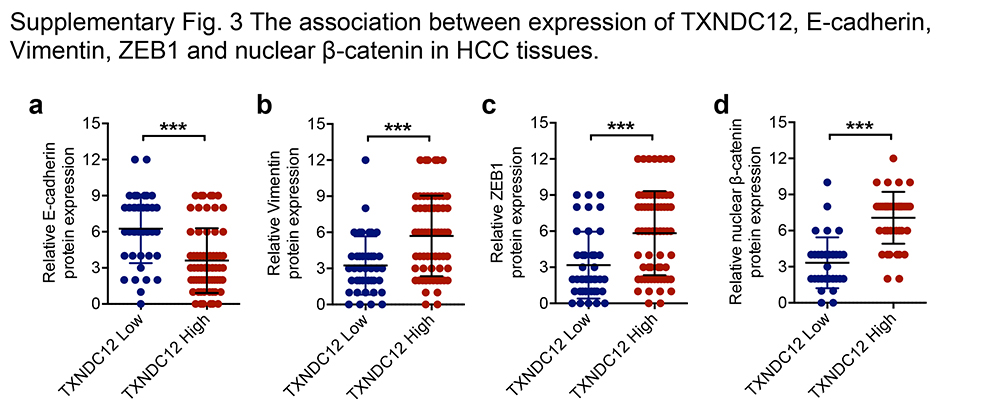
**

**Supplementary Fig. 3** The association between expression of TXNDC12 and E-cadherin, Vimentin, ZEB1 or nuclear β-catenin in HCC tissues. ****P* < 0.001.


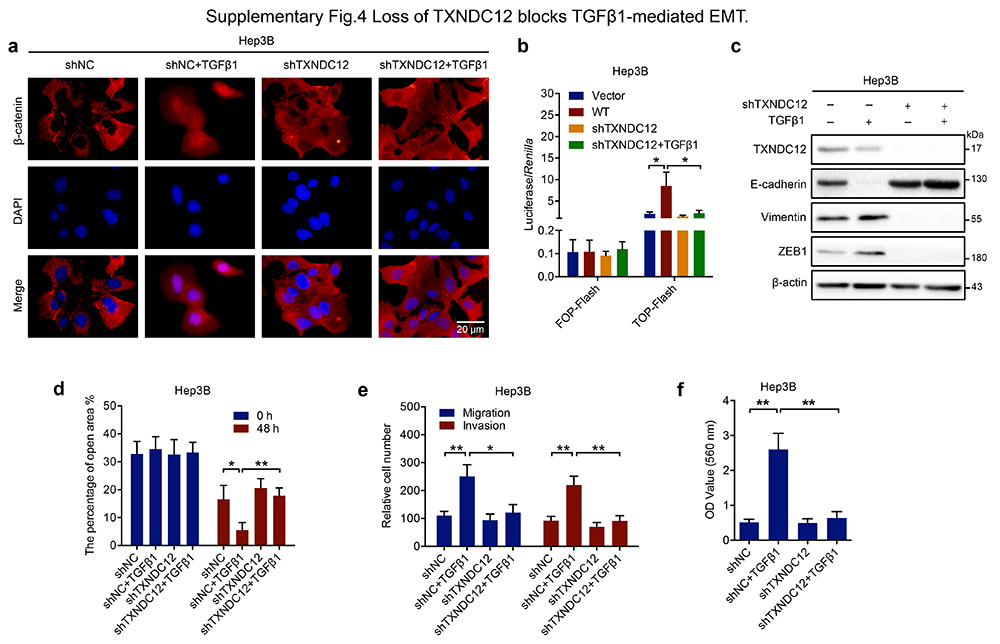


**Supplementary Fig. 4** Loss of TXNDC12 blocks TGFβ1-mediated EMT. **a** β-catenin expression in indicated cells treated with or without TGFβ1 as detected by immunofluorescence assay. Merged images represent overlays of β-catenin (red) and nuclear staining by DAPI (blue); scale bar, 20 mm. **b** TOP-Flash/FOP-Flash assay depicting β-catenin activity in indicated HCC cells treated with or without TGFβ1. **c** Relative expression levels of TXNDC12, E-cadherin, Vimentin and ZEB1 in indicated cells treated with or without TGFβ1. **d** Wound healing migration assays performed with indicated HCC cells treated with or without TGFβ1. Data represent the mean ± SD and are representative of three independent experiments. **e** Transwell migration and Matrigel invasion assays performed with indicated HCC cells treated with or without TGFβ1. Data represent the mean ± SD and are representative of three independent experiments. **f** Representative data of cell adhesion assay performed with indicated HCC cells treated with or without TGFβ1. Data represent the mean ± SD and are representative of three independent experiments. **P* < 0.05; ***P* < 0.01.


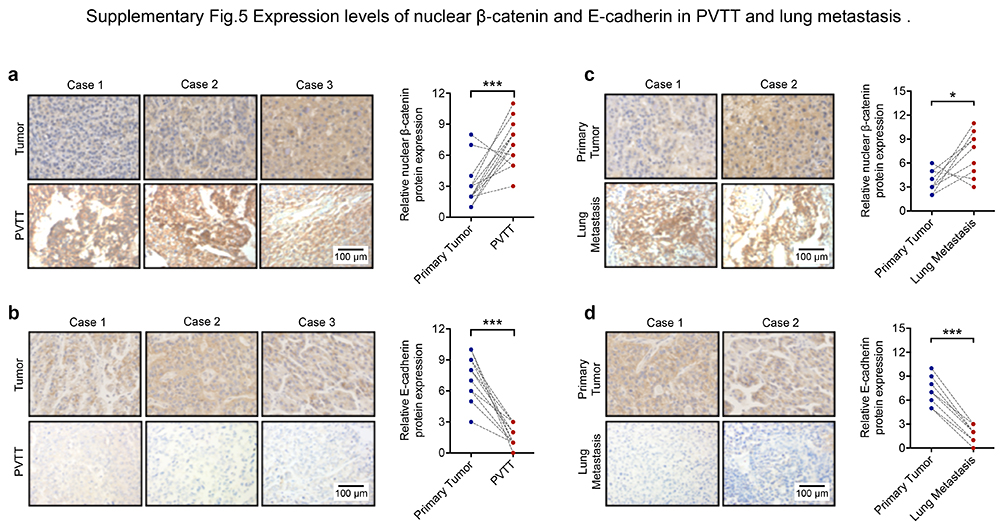


**Supplementary Fig. 5** Expression levels of nuclear β-catenin and E-cadherin in PVTT and lung metastasis. **a, b** Representative images and quantification of nuclear β-catenin (a) and E-cadherin (b) in portal vein tumor thrombus and matched primary HCC tissues obtained by immunohistochemical analysis (n = 14); scale bar, 20 μm. **c, d** Representative images and quantification of nuclear β-catenin (c) and E-cadherin (d) in lung metastases and matched primary HCC tissues obtained by immunohistochemical analysis (n = 10); scale bar, 20 μm. **P* < 0.05; ***P* < 0.01; ****P* < 0.001.

| Supplementary table 1. Demographic data for patients with hepatocellular carcinoma. | | |
| --- | --- | --- |
|  | Men (n = 82) | Women (n = 24) |
| Median Age (years) | 52.5 | 46.0 |
| HBsAg Positive |  |  |
| Yes | 80 | 24 |
| No | 2 | 0 |
| NA | 0 | 0 |
| Ishark score |  |  |
| 1-3 | 13 | 8 |
| 4-6 | 49 | 11 |
| NA | 20 | 5 |
| Grade |  |  |
| High or median | 49 | 12 |
| Low | 32 | 12 |
| NA | 1 | 0 |
| Diameter |  |  |
| ≥ 5 cm | 38 | 13 |
| < 5 cm | 44 | 11 |
| NA | 0 | 0 |
| Number of tumors |  |  |
| Single | 56 | 22 |
| Multiple | 26 | 2 |
| NA | 0 | 0 |
| Capusle |  |  |
| Yes | 36 | 8 |
| No | 45 | 16 |
| NA | 1 | 0 |
| AFP |  |  |
| ≥ 20 ng/mL | 44 | 14 |
| < 20 ng/mL | 38 | 10 |
| NA | 0 | 0 |
| Microvascular invasion |  |  |
| Yes | 32 | 10 |
| No | 50 | 14 |
| NA | 0 | 0 |
| Macrovascular invasion |  |  |
| Yes | 17 | 3 |
| No | 65 | 21 |
| NA | 0 | 0 |
| NA, not available | | |

| Supplementary table 2. Correlation between clinicopathologic features in HCC patients and expression of TXNDC12 and nuclear β-catenin. | | | | | | | |
| --- | --- | --- | --- | --- | --- | --- | --- |
|  | TXNDC12 | | |  | β-catenin | | |
|  | Low (n = 46) | High (n = 60) | *P* |  | Low (n = 37) | High (n = 40) | *P* |
| Age (years) | 57.5 | 48.0 | < 0.001 |  | 53.0 | 51.0 | 0.053 |
| Sex |  |  |  |  |  |  |  |
| Male | 36 | 45 | 0.810 |  | 27 | 29 | 0.963 |
| Female | 10 | 14 |  |  | 10 | 11 |  |
| NA | 0 | 1 |  |  | 0 | 0 |  |
| HBsAg Positive |  |  |  |  |  |  |  |
| Yes | 44 | 60 | 0.103 |  | 36 | 39 | 0.955 |
| No | 2 | 0 |  |  | 1 | 1 |  |
| Ishark score |  |  |  |  |  |  |  |
| 1-3 | 7 | 14 | 0.288 |  | 8 | 6 | 0.440 |
| 4-6 | 28 | 32 |  |  | 19 | 23 |  |
| NA | 11 | 14 |  |  | 10 | 11 |  |
| Grade |  |  |  |  |  |  |  |
| High or median | 36 | 25 | < 0.001 |  | 27 | 15 | 0.001 |
| Low | 10 | 34 |  |  | 9 | 25 |  |
| NA | 0 | 1 |  |  | 0 | 0 |  |
| Diameter |  |  |  |  |  |  |  |
| ≥ 5 cm | 26 | 25 | 0.129 |  | 17 | 23 | 0.311 |
| < 5 cm | 20 | 35 |  |  | 20 | 17 |  |
| Number of tumors |  |  |  |  |  |  |  |
| Single | 40 | 38 | 0.006 |  | 31 | 27 | 0.036 |
| Multiple | 6 | 22 |  |  | 6 | 13 |  |
| Capsule |  |  |  |  |  |  |  |
| Yes | 31 | 13 | < 0.001 |  | 24 | 10 | < 0.001 |
| No | 15 | 46 |  |  | 12 | 30 |  |
| NA | 0 | 1 |  |  | 1 | 0 |  |
| AFP |  |  |  |  |  |  |  |
| ≥ 20 ng/mL | 18 | 40 | < 0.001 |  | 21 | 29 | 0.148 |
| < 20 ng/mL | 28 | 20 |  |  | 16 | 11 |  |
| Microvascular invasion |  |  |  |  |  |  |  |
| Yes | 7 | 35 | 0.003 |  | 30 | 16 | < 0.001 |
| No | 39 | 25 |  |  | 7 | 24 |  |
| Macrovascular invasion |  |  |  |  |  |  |  |
| Yes | 4 | 16 | 0.019 |  | 5 | 12 | 0.081 |
| No | 42 | 44 |  |  | 32 | 28 |  |
| NA, not available | | | |  |  |  |  |

| Supplementary Table 3. Univariable analysis of overall survival and disease free survival. | | | | | |
| --- | --- | --- | --- | --- | --- |
|  | Overall survival | |  | Disease free survival | |
|  | No. of  cases/total | Hazard Ratio |  | No. of  cases/total | Hazard Ratio |
| Sex |  |  |  |  |  |
| Male | 37/82 | 1.00 (Reference) |  | 44/82 | 1.00 (Reference) |
| Female | 8/24 | 0.89 (0.41-1.91) |  | 13/24 | 1.29 (0.70-2.41) |
| Ishark score |  |  |  |  |  |
| 1-3 | 8/21 | 1.00 (Reference) |  | 11/21 | 1.00 (Reference) |
| 4-6 | 29/60 | 1.15 (0.52-2.53) |  | 34/60 | 1.06 (0.54-2.10) |
| Grade |  |  |  |  |  |
| High or median | 17/61 | 1.00 (Reference) |  | 24/61 | 1.00 (Reference) |
| Low | 28/44 | 3.60 (1.96-6.61) |  | 32/44 | 2.95 (1.73-5.04) |
| Diameter |  |  |  |  |  |
| < 5 cm | 20/51 | 1.00 (Reference) |  | 22/51 | 1.00 (Reference) |
| ≥ 5 cm | 25/55 | 1.36 (0.75-2.45) |  | 35/55 | 2.04 (1.19-3.49) |
| Number of tumors |  |  |  |  |  |
| Single | 30/78 | 1.00 (Reference) |  | 40/78 | 1.00 (Reference) |
| Multiple | 15/28 | 1.75 (0.93-3.28) |  | 17/28 | 1.59 (0.90-2.81) |
| Capsule |  |  |  |  |  |
| Yes | 9/44 | 1.00 (Reference) |  | 14/44 | 1.00 (Reference) |
| No | 36/61 | 4.29 (2.05-8.94) |  | 42/61 | 3.67 (1.99-6.75) |
| AFP |  |  |  |  |  |
| < 20 ng/mL | 15/48 | 1.00 (Reference) |  | 17/48 | 1.00 (Reference) |
| ≥ 20 ng/mL | 30/58 | 1.92 (1.03-3.59) |  | 40/58 | 2.72 (1.54-4.80) |
| Microvascular invasion |  |  |  |  |  |
| No | 19/64 | 1.00 (Reference) |  | 27/64 | 1.00 (Reference) |
| Yes | 26/42 | 2.88 (1.59-5.24) |  | 30/42 | 2.84 (1.68-4.80) |
| Macrovascular invasion |  |  |  |  |  |
| No | 32/86 | 1.00 (Reference) |  | 40/86 | 1.00 (Reference) |
| Yes | 13/20 | 2.90 (1.50-5.61) |  | 17/20 | 3.88 (2.16-6.96) |
| TXNDC12 |  |  |  |  |  |
| Low | 11/46 | 1.00 (Reference) |  | 16/46 | 1.00 (Reference) |
| High | 34/60 | 3.80 (1.91-7.54) |  | 41/60 | 3.54 (1.98-6.36) |
| β-catenin |  |  |  |  |  |
| Low | 10/37 | 1.00 (Reference) |  | 15/37 | 1.00 (Reference) |
| High | 24/40 | 3.41 (1.62-7.20) |  | 26/40 | 2.71 (1.43-5.16) |
